# Measuring the Adverse Social Exposome Over the Life Course

**DOI:** 10.1001/jamanetworkopen.2025.12296

**Published:** 2025-05-01

**Authors:** Omonigho M. Bubu, Joshua Gills, Lisa L. Barnes

**Affiliations:** Department of Psychiatry, NYU (New York University) Grossman School of Medicine, New York, New York; Department of Population Health, NYU Grossman School of Medicine, New York, New York; Department of Neurology, NYU Grossman School of Medicine, New York, New York; NYU Alzheimer’s Disease Research Center, NYU Grossman School of Medicine, New York, New York; Department of Psychiatry, NYU (New York University) Grossman School of Medicine, New York, New York; Department of Population Health, NYU Grossman School of Medicine, New York, New York; Department of Neurology, NYU Grossman School of Medicine, New York, New York; Rush Alzheimer’s Disease Center, Rush University Medical Center, Chicago, Illinois

The social exposome represents a diverse set of environmental exposures, including socioeconomic, geographic, cultural, and institutional factors throughout the life course, that influence health and contribute to inequities across populations beyond individual level factors. Research has explored various aspects of the social exposome on health outcomes, noting that a substantial portion of the global disease burden is due to environmental factors and gene-environment interactions.^1^ However, challenges remain in measuring and operationalizing the social exposome, particularly regarding timing, as most studies have been cross-sectional with a single assessment in time. Given that social inequalities can interact with biological factors throughout life and lead to disease over time,^2^ studies that capture the social exposome across the life course to examine timing and the cumulative effects of exposures on health and disease burden are crucial.

Keller et al^3^ examined the association between life course social and environmental exposures, measured by geo-linked time-concordant Area Deprivation Index (ADI) rankings, and the presence or absence of vascular brain injury (VBI) in a sample of brain bank donors from 2 Alzheimer Disease Research Centers (ADRCs). VBI was characterized as infarcts, microinfarcts, hemorrhages, and white matter rarefaction from donations made between 2000 and 2018. In this study, investigators also created measures of the cumulative social exposome for each year of a donor’s lifetime and changes in the social exposome from youth (0–18 years of age) to adulthood (19–64 years of age) to older adulthood (≥65 years of age) and linked this information to well-phenotyped tissue within the existing brain bank data. Using a novel residential history construction method, the study team was able to connect ADIs to 68.1% of person-years, which they summarized for analytic models as both the number of years spent above the population median (ADI, 7.08) and changes across life course periods. Overall, living more years in a county with an ADI above the population median was associated with increased odds of VBI, by about 4%. They also reported that both increases in ADI from youth to adulthood and youth to older adulthood and stable high ADI across life periods was associated with increased odds of VBI. Thus, suggesting that exposure to social and environmental disadvantage over the life course, especially increasing ADI with age (ie, downward mobility), is associated with postmortem cerebrovascular disease.

This study is notable for being the first to date to retrospectively add cumulative social exposome data to well-phenotyped tissue from ADRC brain bank donors. Previous studies have highlighted the rarity of linking life-course exposome data to brain tissue and the tendency of longitudinal social exposome studies to exclude older adults. The social exposome was assessed using geo-linked time-concordant ADI rankings for each year of a donor’s life, with residential histories spanning 1905 to 2018, and including a wide range of older adults, with more than three-quarters being older than 75 years at death. To characterize the social exposome over such a large timeframe for more than 700 donors represents an incredible amount of time and effort. Further, this research marks a pioneering step in investigating whether critical or sensitive periods of adverse social exposome exposure may contribute to poor brain health and demonstrates that both the dosage and timing of exposures may be important.

Despite the noted strengths, some challenges are worth noting. Comparisons of the demographic characteristics across those included and excluded from analyses showed differences across various pertinent demographic subgroups, including age at death, birth year, death year, sex distribution, and racial background. Those who were excluded due to missing data on vascular pathology were more likely to come from the earlier birth cohort (born before 1920) and may not have had the relevant outcome data because of changes in the National Alzheimer Coordinating Center neuropathological data collection forms over time as autopsies accrued over several decades. There was also limited diversity in the autopsy sample, with 79.7% from White decedents and 0.5% from Black or Asian backgrounds—2 groups known to experience high rates of cerebrovascular disease.^4^ In fact, although the ADI values ranged from 1 (least disadvantaged counties) to 100 (most disadvantaged counties), the median value for their sample was just over 7 and relatively homogenous across the life course. Thus, replicating the study within a larger multisite study including more of the ADRC brain bank sites with diverse participants representing a larger range of social disadvantages is necessary. In addition, the authors attempted to address the issue of missingness by using linear interpolation, assuming a linear progression of ADI between 2 nonmissing points. However, few things in life move in straight lines or have constant motion over time. Thus, it may be important to use methods in geospatial literature with a more nuanced approach and that go beyond the linearity assumption.

The authors developed 13 distinct versions of the ADI for each decade from 1900 to the present, using historical census and geographic data to reflect evolving area-level characteristics. While this approach is commendable, harmonizing data across these versions was challenging due to changes in the census each decade, making it impossible to fully replicate the ADI for each period. To address this, life course researchers should focus on key variables such as education, employment, income, poverty, and housing characteristics available across decades to ensure consistent operationalization of the social exposome. The life course was divided into 3 periods: youth, adulthood, and older adulthood, based on literature, the US legal adult age, and typical retirement age. However, considering the focus on residential housing, future studies might benefit from further dividing adulthood into 2 periods: 19 to 39 years of age and 40 to 64 years of age. This division would better capture changes related to housing purchases, job opportunities, and movement patterns across different ages. Finally, the ADI lacks information on variables such as insurance status, work status, drug and alcohol use, or smoking status, which are crucial for assessing the risk of VBI within the broader exposome.

Results of this study suggest that the social exposome, characterized over the life course, is an important risk factor in age-related vascular pathology. Because the social exposome is shaped by broader societal policies and practices, including racism, residential segregation, and neighborhood disinvestment, dismantling the macrosystems and structures that create differences in social exposures for different populations in the first place will be an important step for reducing disparities in ADRD.^5,6^ Further, understanding the mechanisms linking adverse social exposures to poor brain health, such as increased stress and anxiety, poor sleep, and unhealthy behaviors like smoking, alcohol, or substance use, to name a few,^7^ will create opportunities for targeted interventions at critical points in the life course to reduce cerebrovascular disease and prevent ADRD.
